# Comparison of performance between Fast Track Diagnostics Respiratory Kit and the CDC global reference laboratory for influenza rRT‐PCR panel for detection of influenza A and influenza B

**DOI:** 10.1111/irv.12830

**Published:** 2021-02-26

**Authors:** Assana Cissé, Jennifer Milucky, Abdoul Kader Ilboudo, Jessica L. Waller, Brice Bicaba, Isaïe Medah, Sara Mirza, Cynthia G. Whitney, Zekiba Tarnagda

**Affiliations:** ^1^ Laboratoire National de Référence‐Grippes (LNR‐G) Institut de Recherche en Sciences de la Santé (IRSS) Bobo Dioulasso Burkina Faso; ^2^ Centers for Disease Control and Prevention National Center for Immunization and Respiratory Diseases Atlanta GA USA; ^3^ Direction de la Protection de la Santé de la Population Ministère de la Santé Ouagadougou Burkina Faso

**Keywords:** Burkina Faso, diagnostics, influenza, severe acute respiratory infections, West Africa

## Abstract

**Background:**

Reliable diagnostics are a key to identifying influenza infections.

**Objectives:**

Our objectives were to describe the detection of influenza among severe acute respiratory infection (SARI) cases, to compare test results from the Fast Track Diagnostics (FTD) Kit for influenza detection to the Centers for Disease Control (CDC) human influenza virus detection and characterization panel, and to assess seasonality of influenza in Burkina Faso.

**Methods:**

Nasopharyngeal and oropharyngeal specimens from SARI cases (hospitalized patients with fever, cough, and onset in the previous 10 days) were tested using the FTD‐33 Kit and the CDC rRT‐PCR influenza assays. We assessed sensitivity and specificity of the FTD‐33 Kit for detecting influenza A, influenza B, and the influenza A(H1N1)pdm09 strain using the CDC human influenza rRT‐PCR panel as the gold standard.

**Results:**

From December 2016 to February 2019, 1706 SARI cases were identified, 1511 specimens were tested, and 211 were positive for influenza A (14.0%) and 100 for influenza B (6.6%) by either assay. Higher influenza circulation occurred between November and April with varying peaks of influenza A and influenza B. Sensitivity of the FTD‐33 assay was 91.9% for influenza A, 95.7% for influenza B, and 93.8% for A(H1N1)pdm09 subtype. Specificity was over 99% for all three tests.

**Conclusions:**

Our study indicates that Burkina Faso has one peak of influenza each year which is similar to the Northern Hemisphere and differs from other countries in West Africa. We found high concordance of influenza results between the two assays indicating FTD‐33 can be used to reliably detect influenza among SARI cases.

## 1 INTRODUCTION

Influenza viruses cause acute respiratory infections in all parts of the world and can lead to hospitalization and death. An estimated 5%‐10% of adults and 20%‐30% of children worldwide are affected by seasonal influenza each year.^1^ A recent model by Iuliano et al. estimated that 4‐8.8 per 100000 influenza‐associated deaths occur annually around the world, with the highest rates occurring in sub‐Saharan Africa, the western Pacific, and South‐East Asia.^2^ Surveillance for influenza that includes accurate laboratory diagnostics plays a critical role for annual vaccine strain selection, identification of novel strains, and quick recognition of increased influenza circulation that could signal a pandemic. One report on the seasonality of influenza which included influenza‐like illness (ILI) and/or severe acute respiratory infection (SARI) surveillance in eight West African countries described two peaks; however, data included in this report from Burkina Faso did not include SARI cases, and country‐specific seasonality was limited.^3^

Clinical presentation of influenza is similar to that of many other viral, bacterial, and fungal pathogens causing acute respiratory illnesses; thus, diagnostics play a key role in identifying which acute respiratory infections are caused by influenza. The Centers for Disease Control and Prevention (CDC) reverse transcription quantitative PCR (rRT‐PCR) assay for detection and characterization of influenza^4^ is utilized in influenza reference laboratories around the world and is considered a reliable standard for influenza testing by the World Health Organization.^5^ Respiratory illnesses are caused by a variety of pathogens, and incorporating multi‐pathogen diagnostic methods into respiratory disease surveillance platforms can help ministries of health determine potential causes of outbreaks or know when respiratory pathogens are circulating. Fast Track Diagnostics Respiratory Pathogens 33 Respiratory (FTD‐33) Kit is a multi‐pathogen platform using real‐time reverse‐transcriptase polymerase chain reaction (rRT‐PCR) to detect 33 pathogens in humans with acute respiratory illness.^6^ It is important that the individual targets in a multipathogen platform perform well compared with single‐target assays.

Burkina Faso is a sub‐Saharan country in West Africa with a population of approximately 20 million people. One of the leading causes of death is lower respiratory tract infections, which account for more than 14% of deaths annually.^7^ The country implemented a sentinel surveillance system of ILI in 2010 and in late 2016, began conducting SARI sentinel surveillance to better understand respiratory pathogens circulating and their disease burden among the community. The country has a national influenza reference laboratory with established capacity to conduct influenza testing using the CDC panel and multipathogen testing using the FTD‐33 Kit.^6^ Our objectives were to describe the circulation of influenza among SARI cases in Burkina Faso and to determine sensitivity and specificity of the FTD‐33 Kit for influenza detection compared with the CDC human influenza rRT‐PCR detection and characterization panel.

## 2 METHODS

### 2.1 Study sites and data collection

SARI sentinel surveillance was conducted among inpatients of all ages at one national teaching hospital (Centre Hospitalier Universitaire de Bogodogo [Bogodogo CHU]) and three district hospitals: Boussé Centre Médical avec Antenne Chirurgicale de Kongoussi (Kongussi CMA), Centre Médical avec Antenne Chirurgicale de Houndé (Houndé CMA), and Centre Médical avec Antenne Chirurgicale de Boussé (Boussé CMA). Bogodogo CHU is a large hospital located in the capital city of Ouagadougou. Boussé CMA is located in the Plateau‐Central region; Kongoussi CMA is located in the Centre‐Nord Region; and Houndé CMAs located in the Hauts‐Bassins Region on the western side of the country. Cases were identified using the World Health Organization SARI case definition (an acute respiratory infection with history of fever or measured fever ≥38°C and cough, with onset within the last 10 days, and requiring hospitalization). Trained hospital staff identified cases 7 days a week throughout the study time frame and completed a report form for each identified case that included information on demographics, symptoms of the illness, treatment administered during the hospitalization, and outcome.

Nasopharyngeal (NP) and oropharyngeal (OP) specimens were collected from SARI cases within 24 hours of hospitalization when possible and placed together into one tube of Universal Transport Media (Copan Diagnostics). Only NP swabs were collected from children <6 months of age. Immediately following collection, specimens were refrigerated at four degrees Celsius for temporary storage. Specimens were then transported on cold packs to the national reference laboratory within 72 hours of specimen collection. Upon arrival at the national reference laboratory, specimens were aliquoted and either tested immediately or stored at −80 degrees Celsius until testing could be completed.

#### 2.1.1 Laboratory testing methods

##### Nucleic acid extraction

Total nucleic acid (TNA) was extracted from 400 µL of Universal Transport Media containing the NP and OP specimens and eluted into 110 µL using the EZ1 Advanced XL Instrument with the EZ1 Mini Viral 2.0 Kit (Qiagen) following the manufacturer's instructions. Seven specimens were extracted at a time with an extraction control consisting of nuclease‐free water.

##### Respiratory viruses, bacteria, and fungi real‐time RT‐PCR detection

The TNA extracts from NP/OP specimens were screened individually for detection of respiratory pathogens using eight multiplex reverse‐transcriptase real‐time polymerase chain reactions (rRT‐PCR) from the FTD‐33 Test Kit (Fast Track Diagnostics). The kit is used for detection of the following respiratory viruses, bacteria, and fungi: influenza A, influenza A subtype A(H1N1)pdm09, influenza B, and influenza C; parainfluenza viruses 1, 2, 3, and 4; coronaviruses NL63, 229E, OC43, and HKU1; human metapneumoviruses A and B; rhinovirus; respiratory syncytial viruses A and B; adenovirus; enterovirus; parechovirus; bocavirus; *Pneumocystis jirovecii*; *Mycoplasma pneumoniae*; *Chlamydia pneumoniae*; *Streptococcus pneumoniae*; *Haemophilus influenzae*; *Haemophilus influenzae* type B; *Staphylococcus aureus*; *Moraxella catarrhalis*; *Bordetella* species (excluding *Bordetella parapertussis*); *Klebsiella pneumoniae*; *Legionella* species; and *Salmonella* species.^6^

The eight multiplex rRT‐PCR reactions were set up following the manufacturer's instructions.^6^ Each reaction mix consisted of 10 µL TNA, 1.5 µL of oligonucleotide mix, 12.5 µL 2 X AgPath‐ID^TM^ One‐Step RT‐PCR buffer and 1 µL 25 X AgPath‐ID^TM^ One‐Step RT‐PCR Enzyme Mix (Thermo Fisher Scientific). The manufacturer's instructions recommend introducing exogenous internal control material provided with the FTD‐33 Kit into each clinical specimen prior to nucleic acid extraction and testing with corresponding rRT‐PCR oligonucleotide mix to monitor for PCR inhibition. This step was excluded to minimize potential contamination of primary specimen material. Instead, each specimen was tested in parallel for the presence of human ribonuclease P (RNase P). Detection of RNase P, which is ubiquitous in human cells, serves as a control for specimen extraction and PCR inhibition without manipulation of primary specimens. Reaction mixtures containing a no‐template control, extraction control, and positive control (Resp21 PC or Resp33 PC2) were included for each multiplex reaction mix. An extraction control and no‐template control materials were included for testing of the eight multiplex reaction mixes and the RNase P reaction mix. The positive control (Resp21 PC or Resp33 PC2) consisting of pooled plasmids^6^ from the FTD‐33 Kit was also included for testing of the multiplex reaction mixes and human DNA was included as the positive control for the RNase P reaction mix. All assays were tested using the Applied Biosystems 7500 Real‐Time PCR Instrument (Thermo Fisher Scientific) with the following cycling conditions: 42°C for 15 minutes, 94°C for 3 minutes, 40 cycles of 94°C for 8 seconds, and 60°C for 34 seconds. Any assay or specimen with a control result deviating from the expected result was retested.

##### Human influenza virus real‐time RT‐PCR detection

Influenza viruses were detected using singleplex reverse‐transcriptase real‐time polymerase chain reaction (rRT‐PCR) methods from the Centers for Disease Control and Prevention (CDC) Influenza Division (Atlanta, GA, USA) for typing and subtyping of influenza A and influenza B.^4^ Samples were first screened for influenza A and B viruses. The typing kit includes influenza A, influenza B, and human RNase P primers and probes. Specimens found to be positive for influenza A virus were subtyped using an influenza A(H3/H1pdm09) panel. For influenza B virus–positive samples, an influenza B lineage genotyping panel including B/Victoria and B/Yamagata primers and probes was used. Each reaction mix consisted of 5 µL total nucleic acid, 5 of nuclease‐free water, 0.5 µL of each primer (forward and reverse) and probe, 12.5 µL 2 X AgPath‐ID^TM^ One‐Step RT‐PCR buffer, and 1 µL 25 X AgPath‐ID^TM^ One‐Step RT‐PCR enzyme mix (Thermo Fisher Scientific). Reaction mixtures containing a no‐template control (negative control), extraction control, human specimen control, and pooled influenza‐positive control were included for each singleplex reaction mix. The rRT‐PCR testing was performed at a final volume of 25 µL on the Applied Biosystems 7500 Real‐Time PCR Instrument with the following cycling conditions: 50°C for 30 minutes, 95°C for 10 minutes, 40 cycles of 95°C for 15 seconds, and 55°C for 30 seconds. Any assay or specimen with a control result deviating from the expected result was retested.

### 2.2 Statistical analysis

We described the basic demographics of SARI cases and the frequency of influenza A‐ and influenza B–positive results among SARI cases. Differences in characteristics between groups were assessed using chi‐square, with *P*‐values <.05 considered statistically significant. We also assessed sensitivity and specificity of the FTD‐33 Kit for detecting influenza A, influenza B, and the influenza A(H1N1)pdm09 strain by comparing results of the FTD‐33 Kit to those of the CDC rRT‐PCR panel, considering the CDC rRT‐PCR panel as the gold standard. Other influenza subtypes are unavailable for comparison in the FTD‐33 Kit, and influenza C was not tested by CDC rRT‐PCR panel. We also assessed sensitivity and specificity, limiting the cases to those that were collected during months in which there were greater than three specimens positive for influenza A, influenza B, or influenza A A(H1N1)pdm09 by either laboratory method. For influenza A, influenza B, and the H1N1 assays, we assessed cycle threshold (ct) values of discordant and concordant results. Analysis of concordant positive results relied on the reported FTD‐33 ct value, and analysis of the discordant results relied on the ct value of the positive assay. We conducted chi‐square tests to compare the proportion of concordant and discordant results with ct values above and below 30 for the influenza A, influenza B, and H1N1 assays. *P*‐values <.05 were considered statistically significant. Statistical analyses were conducted using SAS version 9.4 (SAS Institute Inc).

## 3 RESULTS

From December 2016 to February 2019, 1706 SARI cases were identified across the four sentinel surveillance sites. Among these, 1511 (88.6%) had specimens with available test results for both the FTD‐33 and the CDC singleplex assays at the national reference laboratory and were included in this analysis; 30.7% were specimens from Houndé, 30.2% from Kongoussi, 24.3% from Bogodogo, and 14.8% from Boussé (Table 1). Most cases (85.2.%) were in children <5 years of age, and more than half (51.9%) were those in between the ages of 1‐4 years. Influenza A and influenza B detections among SARI cases occurred more often in older age groups. Cases among persons 5 years of age and older had the highest proportion of influenza A (18.1%) or influenza B (9.1%) detected and cases among infants <1 year of age had the lowest proportion of influenza detections (influenza A 10.3%; influenza B 3.7%) (influenza A *P* < .05; influenza B *P* < .05). The proportion of samples with influenza detected did not differ by site.

**TABLE 1:** Influenza A and influenza B detections^a^ among patients (N = 1511) with severe acute respiratory infections by site and age group, December 2016‐February 2019.

	Total (n = 1511)		Flu A positive (n = 211)			Flu B positive (n = 100)		
	N	%	N	%	*P* value	N	%	*P* value
Site								
Bogodogo	367	24.3%	54	14.8%	.612	24	6.6%	.459
Boussé	223	14.8%	25	11.2%		13	5.8%	
Houndé	464	30.7%	68	14.7%		26	5.7%	
Kongoussi	457	30.2%	64	14.1%		37	8.1%	
Age^b^								
<1 y	486	32.2%	50	10.3%	.009	18	3.7%	.005
1‐<5 y	785	51.9%	117	15.0%		61	7.8%	
5 and older	220	14.6%	40	18.2%		20	9.1%	

^a^ A specimen was considered positive if positive by either FTD‐33 or the CDC assay.

^b^ Age was missing for 20 individuals.

**FIGURE 1:**
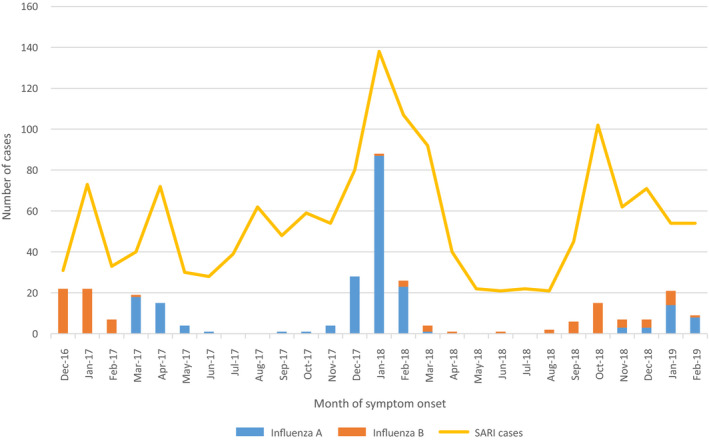
Number of severe acute respiratory infections (SARI) cases and number of SARI cases that tested positive (by any assay) for influenza A and influenza B by month and year of symptom onset, December 2016‐February 2019

SARI case enrollment peaked in January 2018 with 138 cases across all sites during that month (Figure 1); 87 (63.0%) of those cases tested positive for influenza A by any assay. In general, enrollment of SARI cases was higher during the months of October to March than during other months. Influenza B was circulating at the start of our surveillance in December 2016 and influenza A subtype H1N1 began circulating in March 2017. The second respiratory season had very little influenza B circulation, but there were large peaks of influenza A H1N1 circulation beginning in September 2017 and with a marked increase during December 2017‐February 2018. The third respiratory season began with influenza B circulating in August of 2018 followed by influenza A H3N2 circulation in November. As influenza A circulation increased, influenza B circulation decreased. During the months of May to August in 2017 and 2018, very little influenza was detected among our SARI case population.

Overall, 327 (21.6%) specimens were positive for influenza either by FTD‐33 or CDC rRT‐PCR panel, with 211 (14.0%) positive for influenza A, 100 (6.6%) positive for influenza B, and 16 (1.1%) positive for influenza C (testing available by FTD‐33 only; Table 2). All specimens positive for influenza A or influenza B by CDC rRT‐PCR panel were subtyped. Most influenza A–positive specimens were virus subtype A(H1N1)pdm09 (81.5%), and most influenza B–positive specimens were virus subtype Victoria lineage (65.0%).

**TABLE 2:** Results of influenza testing using FTD‐33 and the CDC influenza singleplex assays among 1511 specimens from patients with severe acute respiratory infections, December 2016‐February 2019

	FTD‐33 positive		CDC singleplex assay positive		Positive by either assay	
	N	%	N	%	N	%
Influenza A	195	12.9%	198	13.1%	211	14.0%
Subtype A(H1N1)pdm09	162	10.8%	161	10.7%	172	11.4%
Subtype H3N2	NA	NA	37	2.5%	37	2.5%
Influenza B	96	6.4%	92	6.1%	100	6.6%
Subtype Victoria	NA	NA	65	4.3%	65	4.3%
Subtype Yamagata	NA	NA	25	1.7%	25	1.7%
Influenza C	16	1.1%	NA	NA	16	1.1%

NA ‐ Not applicable

Using the CDC rRT‐PCR panel as the gold standard, sensitivity of the FTD‐33 assay was 91.9% for influenza A, 95.7% for influenza B, and 93.8% for A(H1N1)pdm09 subtype (Table 3). Specificity was over 99% for all three tests. Sensitivity and specificity of the FTD‐33 assay were similar when including only months where influenza was circulating.

**TABLE 3:** Sensitivity and specificity of FTD‐33 influenza assays compared to the CDC influenza assays from all study months (December 2016‐february 2019) and during only those months with influenza circulating (>3 positive tests)

Results from singleplex assay	N tested	FTD+	FTD‐	Sensitivity	Specificity	Confidence intervals	*P* value
All months							
Influenza A positive	198	182	16	91.9%		0.87‐0.96	<.0001
Influenza A negative	1308	13	1295		99.0%	0.98‐0.99	<.0001
Influenza B positive	92	88	4	95.7%		0.89‐0.99	<.0001
Influenza B negative	1412	8	1404		99.4%	0.98‐0.99	<.0001
A(H1N1)pdm09 positive	161	151	10	93.8%		0.89‐0.97	<.0001
A(H1N1)pdm09 negative	1343	11	1332		99.2%	0.98‐0.99	<.0001
Months with influenza circulating^a^							
Influenza A positive	190	175	15	92.1%		0.87‐0.96	<.0001
Influenza A negative	457	11	446		97.6%	0.96‐0.98	<.0001
Influenza B positive	79	76	3	96.2%		0.89‐0.99	<.0001
Influenza B negative	427	8	419		98.1%	0.96‐0.99	<.0001
A(H1N1)pdm09 positive	184	174	10	94.6%		0.89‐0.97	<.0001
A(H1N1)pdm09 negative	362	11	351		97.0%	0.95‐0.99	<.0001

^a^ Results for all specimens during a month were included if >3 specimens tested positive for the target of interest (eg, influenza A, influenza B, or H1N1) with the singleplex assay.

Testing identified 29 specimens with discordant results (positive by one assay and negative by the other) for influenza A results, 12 specimens with discordant influenza B results, and 21 specimens with discordant A(H1N1)pdm09 subtype. All specimens with discordant results arrived at the laboratory in good condition (i.e., frozen), and there was no difference in discordant results across site or age‐group. Specimens with discordant results were significantly more likely to have a ct value above 30 than those with concordant results for all three assays (*P* < .05; Table 4). Analysis of concordant positive results relied on the reported FTD‐33 ct value, and analysis of the discordant results relied on the ct value of the positive assay. For the influenza A and influenza A(H1N1)pdm09 subtype assays, discordant results were equally likely to be positive for both assays; however, for influenza B, discordant results were more frequently positive with the FTD‐33 Kits (8 of 12 discordant results).

**TABLE 4:** Cycle threshold (ct) values ≥30 among specimens with concordant and discordant positive results cases for influenza A, influenza B, and H1N1 subtype by FTD‐33 and CDC influenza assays

	Concordant results^a^			Discordant results^b^			*P* value
	Number of specimens with concordant results	Number of specimens with ct value ≥30	Percent of specimens with ct value ≥30	Number of specimens with discordant results	Number of specimens with ct value ≥30	Percent of specimens with ct value ≥30	
Influenza A	182	44	24.2%	29	18	62.1%	<.05
Influenza B	88	17	19.3%	12	9	75.0%	<.05
A(H1N1)pdm09	151	55	36.4%	21	16	76.2%	<.05

^a^ Analysis of concordant positive results relied on the reported FTD‐33 ct value.

^b^ Analysis of the discordant results relied on the ct value of the positive assay.

## 4 DISCUSSION

Our study found that the results for influenza A, influenza B, and A(H1N1)pdm09 assays for FTD‐33 and the CDC rRT‐PCR panel were similar. In particular, concordance of testing results was very good when there was a higher viral load (lower ct value) in the specimen. We reported sensitivity of greater than 93% and specificity of greater than 99% for all three assays. Other studies have reported on sensitivity and specificity or concordance of the FTD‐33 assay compared with either other multipathogen platforms or in‐house singleplex tests.^8^, ^9^, ^10^, ^11^, ^12^, ^13^ Most of these studies reported comparable results for the influenza A, influenza B, and H1N1 assays; however, two reported significant discrepancies with the influenza B assay.^8^, ^10^ Most specimens with discordant results had higher ct values, closer to the limit of detection; thus, discordant results were not unexpected given the lower concentrations of virus in the specimens. We did not find that these results differed by age‐group or site, and all discordant specimens arrived in the laboratory in good condition.

We also found that in Burkina Faso, a country with distinct dry and rainy seasons, influenza did not circulate year‐round, but rather circulated primarily during the same months as influenza circulation in Northern Hemisphere countries. A publication summarizing influenza‐like illness and SARI surveillance data across eight West African countries, including Burkina Faso, reported year‐round circulation of influenza with two distinct peaks of circulation (January‐March and August‐November) which differs from our findings.^3^ However, over the 3‐year study period, only 1009 influenza‐like illness cases and no SARI cases from Burkina Faso were included in that analysis limiting the overall contribution of cases from Burkina Faso included in the analysis. Additionally, overall percent of influenza positives was much higher in our study which may indicate there are differences in individuals captured using an influenza‐like illness case definition vs a SARI case definition. An understanding of seasonality of influenza within the country can inform seasonal influenza vaccine policy and pandemic preparedness. Further assessment of the surveillance system will allow the country to identify groups at risk for influenza and can inform clinical management of cases. Additionally, use of the FTD‐33 assay for SARI surveillance allows for detection of other pathogens when influenza is not detected as well as co‐detections with influenza.

There are some limitations to our study. Our comparisons of influenza results were limited to influenza A, influenza B, and A(H1N1)pdm09 subtype. There is no FTD‐33 assay for influenza A subtype H3N2 or influenza B lineages for comparison, and there was no CDC assay for influenza C available. Study staff were trained multiple times on specimen collection techniques; however, during busy respiratory months the clinical staff may have missed SARI cases due to other clinical responsibilities which may have implications for the number of influenza cases identified during peak seasons. We were unable to include detection of additional respiratory pathogens in this report. A further investigation of additional pathogen detections would provide additional information about other respiratory pathogens circulating in this population.

## 5 CONCLUSION

FTD‐33 allows for rapid detection of 33 respiratory pathogens, and laboratorians were able to conduct the complex testing in a timely and efficient manner that provided accurate test results for detection of influenza. Additionally, multiplex methods reduce the amount of specimen volume needed to test for the large number of pathogens. However, management of data from multipathogen platforms remains a challenge, especially for surveillance purposes. Our study showed similar results between FTD‐33 and the CDC rRT‐PCR panel and highlights a seasonality of influenza circulation similar to that of the Northern Hemisphere during the 27 months of SARI case enrollment. Further assessment of epidemiologic and clinical characteristics of influenza cases will allow for public health officials to better understand risk groups and implement vaccine policies.

## CONFLICT OF INTEREST

The authors have no potential conflicts to disclose.

## DISCLAIMER

The findings and conclusions in this report are those of the authors and do not necessarily represent the official position of the Centers for Disease Control and Prevention.

## AUTHOR CONTRIBUTION

**Assana Cisse:** Data curation (equal); Formal analysis (equal); Investigation (equal); Project administration (equal); Software (equal); Validation (equal); Writing‐review & editing (equal). **Jennifer Milucky:** Conceptualization (lead); Data curation (lead); Formal analysis (lead); Funding acquisition (supporting); Investigation (equal); Methodology (equal); Project administration (equal); Resources (supporting); Software (equal); Supervision (equal); Validation (lead); Visualization (lead); Writing‐original draft (lead); Writing‐review & editing (lead). **Abdoul K. Ilboudo:** Data curation (equal); Investigation (equal); Project administration (equal); Software (equal); Validation (equal); Writing‐review & editing (equal). **Jessica Waller:** Data curation (equal); Formal analysis (supporting); Investigation (equal); Methodology (equal); Project administration (equal); Resources (supporting); Validation (supporting); Writing‐original draft (supporting); Writing‐review & editing (equal). **Brice Bicaba:** Conceptualization (supporting); Data curation (supporting); Funding acquisition (supporting); Investigation (supporting); Project administration (supporting); Resources (supporting); Supervision (equal); Writing‐review & editing (equal). **Isaïe Médah:** Conceptualization (supporting); Funding acquisition (equal); Resources (equal); Supervision (equal); Writing‐review & editing (equal). **Sara A Mirza:** Conceptualization (equal); Funding acquisition (lead); Investigation (equal); Project administration (equal); Resources (equal); Supervision (equal); Writing‐review & editing (equal). **Cynthia Whitney:** Conceptualization (equal); Investigation (equal); Methodology (equal); Resources (supporting); Supervision (equal); Writing‐review & editing (equal). **Zekiba Tarnagda:** Conceptualization (supporting); Funding acquisition (supporting); Investigation (equal); Methodology (equal); Resources (supporting); Supervision (lead); Writing‐review & editing (equal).

## Data Availability

Data may be available pending approval from the Burkina Faso Ministry of Health.
